# Assessment of dietary spirulina supplementation on growth performance, nutrient digestibility, and intestinal health in lipopolysaccharide-challenged weanling pigs

**DOI:** 10.1093/jas/skaf338

**Published:** 2025-09-27

**Authors:** Emmanuel O Alagbe, Kolapo M Ajuwon, Hagen Schulze, Olayiwola Adeola

**Affiliations:** Department of Animal Sciences, Purdue University, West Lafayette, IN 47907; Department of Animal Sciences, Purdue University, West Lafayette, IN 47907; Livalta, an AB Agri Company, Peterborough, UK; Department of Animal Sciences, Purdue University, West Lafayette, IN 47907

**Keywords:** anti-inflammation, antioxidation, growth performance, intestinal health, lipopolysaccharide, spirulina

## Abstract

The intestinal health of weanling pigs is often compromised by harmful bacterial agents, leading to inflammation and reduced intestinal integrity. Limited research exists on the effects of spirulina as a bioactive feed additive on weanling pigs. Hence, this study aimed to investigate the effect of spirulina on the growth performance and intestinal health of pigs using a lipopolysaccharide (LPS) challenge model. On d 0, 128 pigs were assigned to one of four groups in a 2 × 2 factorial arrangement of treatments with 2 levels each of spirulina (0 or 5 g SP/kg) and LPS (challenge or no-challenge). There were 8 replicate pens per treatment and 4 pigs per pen. On d 7, pigs were weighed, and pigs in the LPS challenge group were intraperitoneally injected with 100 µg/kg body weight LPS. The other pigs were injected with saline. On d 14, all pigs were weighed. On d 15, one selected pig per pen was again injected with the same amount of LPS or saline. After 4 h, the selected pigs were euthanized, and samples were collected. Data were analyzed using the MIXED procedure of SAS and evaluated for main effects and interactions. There was no dietary spirulina effect on growth performance. However, the LPS challenge reduced (*P *< 0.05) the body weight and gain-to-feed ratio of pigs. Dietary spirulina increased (*P *< 0.05) the apparent total tract digestibility (ATTD) of dry matter regardless of the LPS challenge. There was an SP × LPS interaction effect (*P *< 0.05) on the jejunal villus height, villus perimeter, and villus area. Additionally, there was an SP × LPS interaction (*P *< 0.05) on serum tumor necrosis factor alpha. Challenge with LPS reduced (*P *< 0.05) the serum concentration of catalase but increased (*P *< 0.05) serum c-reactive protein concentration irrespective of dietary spirulina inclusion level. Dietary spirulina increased (*P *< 0.05) the mRNA expression of jejunal zonula occludens-1. An SP × LPS interaction (*P *< 0.05) showed that LPS-challenged pigs fed a spirulina-supplemented diet had the highest jejunal superoxide dismutase 2 mRNA expression relative to pigs in the other groups. In summary, dietary spirulina supplementation enhanced antioxidant activity and reduced intestinal inflammation in pigs during an LPS challenge. Dietary spirulina also improved tight junction integrity and dry matter digestibility, irrespective of the LPS challenge.

## Teaser

We evaluated the effect of dietary spirulina supplementation in weanling pigs under immune stress, exploring its potential to improve intestinal integrity, enhance antioxidant capacity, and mitigate inflammation. The findings support spirulina as a promising bioactive feed additive for pigs and highlight the need for further research on its long-term effects on growth performance.

## Introduction

Exposure to harmful bacterial-derived agents in the environment poses a significant challenge to the intestinal health of weanling pigs, often leading to infection and inflammation. These disruptions can impair growth performance, compromise intestinal integrity, increase intestinal permeability, and negatively impact nutrient absorption (Liu et al., 2008; Xue et al., 2021). Lipopolysaccharide (LPS), an endotoxin from gram-negative bacteria, is a major contributor to these adverse effects in weanling pigs (Mani et al., 2013). It activates toll-like receptor 4, triggering inflammation, compromising intestinal integrity, increasing oxidative stress, reducing nutrient absorption, and ultimately impairing growth performance (Lu et al., 2008; He et al., 2019; Prates et al., 2021). As a result, the LPS challenge model is well-established in animal research (Xue et al., 2021) to investigate intestinal health challenges and develop potential nutritional mitigation strategies in weanling pigs.

Considerable interest has been directed towards nutritional interventions aimed at improving intestinal integrity and health in weanling pigs, with the goal of modulating the complex internal and external influences affecting pigs during the post-weaning period. One such strategy is the dietary supplementation of spirulina (*Arthrospira platensis*) in pig diets. Spirulina is a filamentous blue-green algae with a nutritional profile that includes phycocyanin, polysaccharides, essential amino acids, B vitamins, polyunsaturated fatty acids, minerals, flavonoids, polyphenols, and carotenoids (Finamore et al., 2017; Fries-Craft et al., 2021). These components, particularly phycocyanin, flavonoids, and carotenoids, exhibit anti-inflammatory, antioxidant, and immunomodulatory properties (Han et al., 2021). For instance, spirulina supplementation downregulated LPS-induced upregulation of tumor necrosis factor alpha (TNFα), cyclooxygenase-2, and interleukin 6 (IL6) genes in BV-2 microglial cells (Chen et al., 2012). Additionally, spirulina protected SH-SY5Y neuroblastoma cells from iron-induced toxicity by preserving the activities of antioxidant enzymes such as glutathione peroxidase and glutathione reductase (Bermejo-Bescós et al., 2008). Furthermore, its anti-inflammatory effects have been demonstrated through the suppression of interleukin 1 beta (IL1β) and TNFα activity via inhibition of the nuclear factor kappa B (NF-κB) signaling pathway (Finamore et al., 2017). These properties position spirulina as a promising feed additive with the potential to mitigate the negative effects of an LPS challenge on pig health.

To the best of our knowledge, there is limited information regarding the effects and possible mode of action of dietary spirulina on ameliorating the negative effects of an LPS challenge on growth performance, nutrient digestibility, and intestinal health of weanling pigs. Therefore, the objective of the current study was to test the hypothesis that dietary spirulina supplementation would ameliorate the LPS-induced disruption in the growth performance, nutrient digestibility, jejunal morphology, intestinal integrity, and immune status of weanling pigs.

## Materials and Methods

The experimental protocol for the current study was reviewed and approved by the Purdue University Animal Care and Use Committee (West Lafayette, IN) under protocol number 1111000250.

### Animals, diets, experimental design, and lipopolysaccharide challenge

A total of 128 newly weaned pigs (Duroc × Landrace × Yorkshire) with an initial average body weight (BW) of 7.39 ± 0.61 kg (64 barrows and 64 gilts; 28 d of age) were used for this experiment. On d 0, pigs were randomly assigned to 4 experimental groups in a 2 × 2 factorial arrangements. The factorial arrangement consisted of two levels of spirulina (0 or 5 g/kg) and LPS challenge (no-challenge or challenge) in a randomized complete block design with d 0 BW used as a blocking factor.

In greater detail, the four experimental treatment groups included: 1) 0 g/kg dietary spirulina with no LPS challenge, 2) 0 g/kg dietary spirulina with LPS challenge, 3) 5 g/kg dietary spirulina with no LPS challenge, and 4) 5 g/kg dietary spirulina with LPS challenge. Each of the four treatment groups consisted of eight replicate pens (four pens of barrows and four pens of gilts), with four pigs per replicate pen. The experimental diets were corn-soybean meal-based and were provided in mash form (Table 1). Pigs were given *ad libitum* supply of feed and water throughout the study. All experimental diets were prepared to meet the nutrient requirements of pigs suggested by NRC (2012), and the diets were fed to pigs from d 0 to 15. Titanium dioxide was added at 5 g/kg as an indigestible marker. The spirulina used for this current experiment was obtained from RFI Ingredients (Broomfield, CO, USA), and it was provided in powdered form.

**Table 1.** Ingredient and nutrient composition of diets, as-fed basis^1^

Item	SP, g/kg	
	0	5
Ingredient, g/kg		
Corn	488.3	438.3
Wheat middlings	50.0	50.0
Soybean meal (48% CP)	316.5	316.5
Dried whey	42.0	42.0
Soybean oil	37.0	37.0
Ground limestone	15.0	15.0
Monocalcium phosphate	10.5	10.5
Salt	3.4	3.4
Vitamin premix^2^	2.5	2.5
Mineral premix^3^	0.7	0.7
Selenium premix^4^	0.5	0.5
L-Lysine HCl	5.2	5.2
DL-Methionine	1.2	1.2
L-Threonine	1.8	1.8
L-Valine	0.5	0.5
SP premix^5^ (corn based)	0.0	50.0
Titanium dioxide premix^6^ (corn based)	25.0	25.0
Total	1000.0	1000.0
Calculated nutrient, g/kg		
ME, kcal/kg	3416	3399
Crude protein	206	206
Analyzed nutrient, g/kg		
Gross energy, kcal/kg	4088	4001
Crude protein	197	199

1 SP = spirulina; ME = Metabolizable energy.

2 Provided the following quantities per kg of complete diet: vitamin A, 6,600 IU; vitamin D_3_, 660 IU; vitamin E, 44 IU; menadione, 2.2 mg; riboflavin, 8.8 mg; D-pantothenic acid, 22 mg; niacin, 33 mg; vitamin B_12_, 0.04 mg.

3 Provided the following quantities per kg of complete diet: I, 0.26 mg; Mn, 12.0 mg; Cu, 6.33 mg; Fe, 136 mg; Zn, 104 mg.

4 Provided 0.3 mg Se/kg of complete diet.

5 A 50 g spirulina premix was prepared by mixing 5 g of spirulina powder with 45 g of corn. When included at 50 g/kg in the diet, this premix provided 5 g/kg of spirulina. The spirulina used in this study contained 12 mg/g chlorophyll, 4.5 mg/g carotenoids, 160 mg/g crude phycocyanin, and 650 g/kg crude protein.

6 Prepared as 5 g titanium dioxide added to 20 g corn

On d 7, all pigs in the LPS challenge groups were intraperitoneally injected with 100 µg/kg BW of *Escherichia coli* (serotype O55: B5) LPS (Sigma-Aldrich, Saint Louis, MO, USA) as described by Wright et al. (2000), while the pigs in the no-challenge groups received intraperitoneal injections of 0.9% physiological saline (Sigma-Aldrich, Saint Louis, MO, USA) following the 2 × 2 factorial treatment arrangement. On d 15, the median BW pig from each pen in the LPS challenge groups was again injected with 100 µg/kg BW of LPS, while the median BW pig from each pen in the no-challenge groups received a saline injection, as performed on d 7.

### Growth performance, sample collection, and processing

Individual BW and feed intake per pen were recorded on d 7 and 14 for the determination of average daily gain (ADG), average daily feed intake (ADFI), and gain-to-feed ratio (G:F). Visual assessment of diarrhea was performed daily from d 7 to 14. Diarrhea severity was scored using a system with the following scale: 1 = normal feces, 2 = pasty feces, 3 = semi-liquid feces, and 4 = watery feces. On d 12, 13, and 14, fecal samples were collected per pen from pigs via gentle rectal palpation. The samples collected on these consecutive days were pooled and stored at −20°C until analysis for apparent total tract digestibility (ATTD) of nutrients.

Four hours after the LPS injection on d 15, blood samples were collected from the median BW pig in each pen via the anterior vena cava into non-heparinized tubes. Serum was obtained by centrifugation at 3,000 × *g* for 15 min at 4°C and stored at −80°C until serum enzyme analyses were performed. Subsequently, the median BW pig in each pen was euthanized using a captive bolt. A mid-jejunal segment of 10 cm was excised, flushed with ice-cold 10% phosphate-buffered saline (PBS; VWR International, Radnor, PA, USA), mounted on cardboard (8 cm), and fixed in 10% buffered formalin (VWR International, Radnor, PA, USA) in a 50 mL tube. The distal jejunum was also excised, flushed with ice-cold PBS, and opened longitudinally to expose the lumen. Mucosal scrapings were then collected using glass slides, placed in 1.5 mL tubes containing TRIzol reagent (Invitrogen, Grand Island, NY, USA), and immediately flash-frozen in liquid nitrogen before storage at −80°C for RNA extraction.

### Intestinal histomorphology

Jejunal tissue sections were processed and stained with hematoxylin and eosin by the Purdue Histology and Phenotyping Laboratory. Villus height, crypt depth, villus area, and villus perimeter were measured using an electronic camera (National Optical and Scientific Instruments, Inc., Schertz, TX, USA) and an ImageJ macro (ImageJ open-source software version 1.8), following the method outlined by Alagbe et al. (2022). Villus height was defined as the distance from the villus tip to the crypt mouth, and crypt depth as the distance from the crypt base to the muscularis mucosa. Villus perimeter was measured by manually tracing the villus boundary using the freehand line selection tool in ImageJ. Villus area was determined by outlining the entire villus using the same tool, with ImageJ calculating the enclosed area (Alagbe et al., 2024). The villus height-to-crypt depth ratio (VH:CD) was subsequently calculated. To minimize intra-sample coefficient of variation, ten (10) measurements per sample were averaged for statistical analysis as established by Alagbe et al. (2025).

### Total RNA extraction and reverse transcription

Total RNA was extracted from jejunal mucosa preserved in TRIzol reagent according to the manufacturer’s protocol. RNA concentration and integrity were assessed using a NanoDrop 1000 spectrophotometer (Thermo Fisher Scientific, Waltham, MA, USA) and 1% agarose gel electrophoresis, respectively. Two micrograms of total RNA from each sample were reverse transcribed into cDNA using M-MLV reverse transcriptase (Promega, Madison, WI, USA). The resulting cDNA was diluted ten-fold with nuclease-free water (Ambion, Austin, TX, USA) and stored at −80°C until further use.

### Quantitative real-time PCR analysis

Real-time PCR was performed to quantify the expression of IL1β, IL6, TNFα, interleukin 10 (IL10), solute carrier family 2 member 1 (SLC2A1), solute carrier family 2 member 5 (SLC2A5), zonula occludens-1 (ZO1), claudin 1 (CLD1), occludin (OCLN), glutathione peroxidase 1 (GPX1), and superoxide dismutase type 2 (SOD2) genes using a Bio-Rad CFX thermocycler (Bio-Rad, Temecula, CA, USA). The reactions were carried out with a SYBR Green-based real-time PCR mix (Biotool, Houston, TX, USA) in a total volume of 10 µL. The PCR protocol included an initial incubation step at 95 °C for 3 min, followed by 40 amplification cycles comprising 95 °C for 10 s, annealing at primer-specific temperature for 30 s, and a final 95 °C for 10 s. After the amplification, melt curve analysis was conducted for each gene after the PCR run to ascertain the specificity and purity of the PCR amplification. The primer sequences and annealing temperatures for each gene are detailed in Supplementary Table 1. All samples were analyzed in triplicates, and an acceptable coefficient of variation of ≤5% was established to ensure reproducibility. Relative gene expression levels were quantified using the 2^−ΔΔCt^ method (Livak and Schmittgen, 2001), with glyceraldehyde-3-phosphate dehydrogenase (GAPDH) serving as the reference gene for normalization.

### Serum biomarkers

The activities of serum superoxide dismutase (SOD; Catalog No. MA-SOD-1, RayBiotech, Peachtree Corners, GA, USA), catalase (CAT; Catalog No. MA-CAT-1, RayBiotech, Peachtree Corners, GA, USA), TNFα (Catalog No. ELP-TNFα-1, RayBiotech, Peachtree Corners, GA, USA), and c-reactive protein (CRP; Catalog No. PPE51-K01, Eagle Biosciences, Amherst, NH, USA) were measured as suggested by the manufacturer.

### Chemical analysis

Before analysis, experimental diets and dried fecal samples were homogenized using a centrifugal mill (ZM 200; Retsch GmbH, Haan, Germany). Dry matter (DM) content was determined by drying the ground samples to a constant weight in a forced-air oven (Precision Scientific Co., Chicago, IL, USA) at 105 °C, following AOAC (2006) method 934.01. Gross energy (GE) was analyzed using an isoperibol bomb calorimeter (Parr 6200; Parr Instrument Co., Moline, IL, USA), while nitrogen (N) content was measured via combustion using a TruMac^®^ N analyzer (LECO Corp., St Joseph, MI, USA) in accordance with AOAC (2000) method 990.03. Titanium concentration was determined spectrophotometrically at 410 nm using a microplate reader, based on the methodology described by Myers et al. (2004).

### Calculations and statistical analysis

The ATTD of nutrients was determined using the index method, following the equation described by Adeola (2001):


$$\text{ATTD}\;\%=100—\left[\left({\text{Ti}}_{I}/{\text{Ti}}_{O}\right)\times \left(D_{O}/D_{I}\right)\times 100\right]$$

where Ti_I_ and Ti_O_ represent the titanium concentrations (g/kg DM) in the diet and fecal samples, respectively, and D_I_ and D_O_ represent the nutrient concentrations (g/kg DM) in the diet and fecal samples, respectively.

Statistical analyses were conducted using SAS (version 9.4; SAS Institute, Cary, NC). Data were analyzed using the MIXED procedure, following a randomized complete block design. The BW of pigs on d 0 was regarded as the blocking factor. Outliers, defined as values exceeding ±1.5 times the interquartile range, were excluded from the analysis. The experimental setup was a 2 × 2 factorial arrangements, with the main effects of SP level (0 or 5 g/kg) and LPS challenge (no-challenge or challenge) included as fixed effects. Blocks were treated as random effects. The interactions between SP and LPS challenge were evaluated, and significant interactions were separated using Tukey’s test. The initial BW served as the blocking factor. Diarrhea frequency was analyzed using the LOGISTIC procedure of SAS. Statistical significance and tendency were declared at *P* ˂ 0.05 and 0.05 ≤ *P* ˂ 0.10, respectively. For growth performance, ATTD, and diarrhea scoring, the pen was considered the experimental unit, while for all other analyses, a pig per pen served as the experimental unit.

## Results

There was no SP × LPS interaction on the growth performance of pigs as shown in Table 2. Moreover, there was no dietary spirulina effect on the ADG, ADFI, or G: F of pigs from d 7 to 14. The LPS challenge reduced (*P *< 0.05) the growth performance of pigs regardless of dietary spirulina addition. Table 2 also shows the simple effects of an LPS challenge and dietary spirulina supplementation on nutrient digestibility in pigs on d 14. The dietary supplementation of spirulina increased (*P *< 0.05) the ATTD of DM, regardless of the challenge state of pigs. The LPS challenge reduced (*P *< 0.05) the ATTD of N and DM in pigs regardless of diet. There was no SP × LPS interaction on the diarrhea scores of pigs from d 7 to 14.

**Table 2.** Simple effects of an LPS challenge and dietary spirulina supplementation on the growth performance of pigs, apparent total tract digestibility of nutrients on d 14, and diarrhea scoring from d 7 to 14

Item	SP, 0 g/kg		SP, 5 g/kg			*P*-values		
	No challenge	Challenge	No challenge	Challenge	SEM^1^	SP	LPS	SP × LPS
Growth Performance^2^								
BW, g								
d 7	8.5	8.8	8.8	8.8	0.27	0.496	0.321	0.365
d 14	9.9	9.8	10.5	9.7	0.30	0.214	0.042	0.124
d 7 to 14								
ADG, g/d	197	133	248	132	21.3	0.220	0.001	0.202
ADFI, g/d	396	339	446	355	29.7	0.163	0.004	0.462
G: F, g/kg	496	406	554	369	45.2	0.819	0.005	0.290
Digestibility								
ATTD of DM, %	82.9	82.5	84.7	83.0	0.58	0.033	0.045	0.214
ATTD of gross energy^3^, %	81.5	82.0	83.7	81.5	0.58	0.154	0.161	0.034
ATTD of N, %	80.9	79.1	81.6	79.3	0.92	0.601	0.018	0.753
Diarrhea Scoring	0.41	0.87	0.65	1.10	0.15	0.228	0.513	0.354

1 SEM = Standard error of mean.

Abbreviations: SP = spirulina; LPS = lipopolysaccharide; BW = body weight; ADG = average daily gain; ADFI = average daily feed intake; G:F = gain-to-feed ratio; DM = dry matter; ATTD = apparent total tract digestibility; N = nitrogen.

2 BW on d 7 reflects pre-LPS challenge values.

3 An SP × LPS interaction (*P *< 0.05) was observed for ATTD of gross energy. However, Tukey’s mean separation showed no significant differences

The simple effects of an LPS challenge and dietary spirulina supplementation on jejunal morphology of pigs on d 15 are outlined in Table 3. There was a significant SP × LPS interaction (*P *< 0.05) for jejunal villus height and villus perimeter, such that dietary spirulina supplementation did not reverse the LPS-induced decrease observed for these indices. Furthermore, a significant SP × LPS interaction (*P *< 0.05) was observed for the jejunal villus area, such that the LPS challenge led to a decrease in the villus area of pigs fed a spirulina-supplemented diet, whereas the villus area remained unchanged in pigs fed the 0 g/kg spirulina diet upon LPS challenge. Regardless of dietary spirulina level, the LPS challenge reduced (*P *< 0.05) jejunal VH: CD.

**Table 3.** Simple effects of an LPS challenge and dietary spirulina supplementation on jejunal morphology of pigs on d 15

Item	SP, 0 g/kg		SP, 5 g/kg			*P*-values		
	No challenge	Challenge	No challenge	Challenge	SEM^1^	SP	LPS	SP × LPS
Jejunum, μm								
Basal width, μm	185	178	188	185	8.2	0.534	0.540	0.867
Mid width, μm	140	143	150	154	5.8	0.073	0.566	0.962
Villus height, μm	395^a^	331^b^	432^a^	289^b^	15.5	0.841	0.001	0.009
Crypt depth, μm	331	326	329	305	13.2	0.335	0.236	0.453
VH: CD	1.25	1.05	1.37	1.01	0.07	0.532	0.001	0.224
Villus perimeter, μm	898^a^	784^b^	985^a^	722^b^	32.4	0.648	0.001	0.013
Villus area, mm^2^	0.05^ab^	0.05^bc^	0.06^a^	0.04**^c^**	0.003	0.237	0.001	0.045

1 SEM = Standard error of mean.

a,b,c Least squares means within a row without a common superscript differ at *P *< 0.05.

Abbreviations: SP = spirulina; LPS = lipopolysaccharide; VH: CD = villus height-to-crypt depth ratio

Table 4 shows the simple effects of an LPS challenge and dietary spirulina supplementation on serum antioxidants and inflammatory biomarkers in pigs on d 15. There was a tendency (*P *= 0.074) for SP × LPS interaction on the serum SOD enzyme. Additionally, there was an SP × LPS interaction (*P *< 0.05) on serum TNFα concentrations, such that serum TNFα increased upon LPS challenge in pigs that had no dietary spirulina supplementation. However, with dietary spirulina addition, there was no change in serum TNFα of pigs upon LPS challenge. The LPS challenge reduced (*P *< 0.05) the serum levels of catalase and increased (*P *< 0.05) serum CRP in weanling pigs irrespective of dietary spirulina inclusion level.

**Table 4.** Simple effects of an LPS challenge and dietary spirulina supplementation on serum antioxidants and inflammatory biomarkers in pigs on d 15

Item	SP, 0 g/kg		SP, 5 g/kg			*P*-values		
	No challenge	Challenge	No challenge	Challenge	SEM^1^	SP	LPS	SP × LPS
Serum biomarkers								
SOD, U/mL	1.57	1.30	1.74	1.92	0.12	0.003	0.707	0.074
Catalase, U/mL	69.5	42.0	61.8	42.0	9.19	0.676	0.016	0.678
TNFα, ng/mL	0.50^b^	0.96^a^	0.49^b^	0.53^b^	0.11	0.032	0.015	0.036
CRP, ug/mL	38.1	57.6	37.4	58.6	6.92	0.981	0.002	0.880

1 SEM = standard error of mean.

a,b Least squares means within a row without a common superscript differ at *P *< 0.05.

Abbreviations: SP = spirulina; LPS = lipopolysaccharide; SOD = superoxide dismutase; TNFα = tumor necrosis factor alpha; CRP = C-reactive proteins

Table 5 shows the effects of an LPS challenge and dietary spirulina supplementation on relative mRNA expression of inflammatory, tight junction, nutrient transporters, and antioxidant genes in the jejunal mucosa of weanling pigs on d 15. There was an SP × LPS interaction (*P *< 0.05) on the jejunal mRNA expression of SOD2, such that in pigs with no dietary spirulina supplementation, the mRNA expression of SOD2 was the same regardless of LPS challenge. However, with dietary spirulina inclusion at 5 g/kg, the relative mRNA expression of the antioxidant SOD2 increased upon LPS challenge. Dietary SP supplementation increased (*P *< 0.05) the mRNA expression of ZO1 regardless of the LPS challenge. However, the LPS challenge increased (*P *< 0.05) the expression of TNFα. There was also a tendency for the LPS challenge to reduce (*P *= 0.065) the mRNA expression of CLD1 in the jejunal mucosa of weanling pigs, irrespective of dietary SP level.

**Table 5.** Simple effects of an LPS challenge and dietary spirulina supplementation on the relative mRNA expression of inflammatory, tight junction, nutrient transporters and antioxidant genes in the jejunal mucosa of weanling pigs on d 15

Item	SP, 0 g/kg		SP, 5 g/kg			*P*-values		
	No challenge	Challenge	No challenge	Challenge	SEM^1^	SP	LPS	SP × LPS
Inflammatory markers								
IL1β	1.00	0.62	0.52	1.12	0.34	0.983	0.742	0.161
IL6	1.00	1.09	0.84	0.99	0.16	0.434	0.480	0.860
TNFα	1.00	1.73	0.74	1.31	0.24	0.175	0.013	0.753
IL10	1.00	0.66	0.67	0.60	0.25	0.448	0.407	0.598
Nutrient transporters								
SLC2A1	1.00	0.97	0.83	0.99	0.17	0.640	0.678	0.542
SLC2A5	1.00	0.87	1.02	0.57	0.26	0.570	0.240	0.509
Tight junction markers								
ZO1	1.00	1.01	1.54	1.69	0.26	0.020	0.734	0.768
CLD1	1.00	0.42	0.68	0.48	0.28	0.504	0.065	0.359
OCLN	1.00	0.96	1.46	1.16	0.29	0.258	0.554	0.658
Antioxidants								
GPX1	1.00	0.50	0.50	0.58	0.22	0.339	0.354	0.186
SOD2	1.00^b^	0.99^b^	1.05^b^	2.04^a^	0.23	0.020	0.034	0.032

1 SEM = Standard error of mean.

a,b Least squares means within a row without a common superscript differ at *P *< 0.05.

Abbreviations: SP = spirulina; LPS = lipopolysaccharide; IL1β = interleukin 1 beta; IL6 = interleukin 6; TNFα = tumor necrosis factor alpha; IL10 = interleukin 10; SLC2A1 = solute carrier family 2 member 1; SLC2A5 = solute carrier family 2 member 5; ZO1= zonula occludens-1, CLD1 = claudin 1; OCLN = occludin; GPX1 = glutathione peroxidase 1; SOD2 = superoxide dismutase type 2

## Discussion

Intestinal damage is a well-established effect of an LPS challenge (Liu et al., 2021; Xiao et al., 2025). In a study by Han et al. (2022), intraperitoneal LPS injection reduced duodenal and ileal villus height and the VH: CD in pigs. Similarly, Xiao et al. (2021) observed that an LPS challenge led to an increase in ileal crypt depth and decreased intestinal barrier integrity. Collectively, these findings indicate that LPS induces intestinal damage, which in turn compromises nutrient absorption (Montoro-Huguet et al., 2021) due to a reduction in surface area available for nutrient uptake.

In the current study, LPS challenge led to a decrease in ADG, ADFI, and G: F in pigs, due to its effect on reducing villus height, villus perimeter, and villus area, and consequently reducing DM, and N digestibility. Moreover, the LPS-induced decrease in growth performance aligns with the results of Kang et al. (2014), which demonstrated that ADFI decreased following an LPS challenge, and ADG declined by more than 12%, due to intestinal damage and alterations in intestinal function (Zhu et al., 2017). Furthermore, Liu et al. (2003) reported an LPS-induced reduction in muscle accretion and feed intake in pigs, with these changes likely mediated by pro-inflammatory cytokines as seen in this study. On another note, although the beneficial effects of dietary spirulina on the growth performance of healthy pigs after weaning have been demonstrated (Grinstead et al., 2000), no significant effect was observed in LPS-challenged pigs.

With dietary spirulina supplementation, pigs exhibited higher ATTD of DM regardless of the LPS challenge. This may be attributed to the effect of dietary spirulina in improving the intestinal epithelial barrier, particularly increasing mRNA expression of ZO1. This finding aligns with previous studies highlighting the role of the intestinal epithelial barrier in animal health, as its maintenance supports nutrient digestion, absorption, and transport (Andrews et al., 2018; Zhang et al., 2023).

Furthermore, this study demonstrates the positive antioxidant and anti-inflammatory effects of dietary spirulina supplementation in weanling pigs. An LPS challenge is known to promote the production of reactive oxygen species (ROS), leading to increased DNA damage and lipid peroxidation (Tan et al., 2015; Cao et al., 2018), which negatively affects growth and intestinal development in weanling pigs (Mercer et al., 1996; Guo et al., 2017). The antioxidant properties of dietary spirulina help neutralize free radicals and protect cells from oxidative damage (Ponce-Canchihuamán et al., 2010) by promoting the transcription and production of scavenging SOD enzyme, as observed in this study. Notably, Xu et al. (2022) demonstrated that components of spirulina can modulate the nuclear factor erythroid 2-related factor 2/Kelch-like ECH-associated protein 1 (Nrf2/Keap1) complex, enabling Nrf2 to translocate to the nucleus. Once in the nucleus, Nrf2 binds to antioxidant response elements (AREs), initiating the transcription of antioxidant genes (Jaiswal, 2004). This provides insight into the potential mechanism by which spirulina exerts its antioxidant effects during an LPS challenge in pigs.

Cellular oxidation and inflammation are closely linked biological processes and excess ROS production induced by LPS can cause tissue damage, triggering the recruitment of inflammatory cytokines (Tan et al., 2015; Biswas, 2016). However, phycocyanin, a major component of spirulina, has been shown to suppress pro-inflammatory markers by blocking ROS-mediated activation of inflammatory pathways (Romay et al., 2001; Bi et al., 2022). This is evident with the reduced serum TNFα concentration observed in LPS-challenged pigs fed a spirulina-supplemented diet compared to those without supplementation. This may reflect the role of dietary spirulina in mitigating excessive inflammatory responses. Taken together, these findings further illustrate the interconnected antioxidant and anti-inflammatory benefits of dietary spirulina supplementation (Wu et al., 2016; Han et al., 2021).

In a high-fat diet-induced gut dysbiosis model, spirulina ameliorated intestinal permeability and restored the expression of tight junction proteins, including ZO-1, OCLN, and CLD1, in rats (Yu et al., 2020). Similarly, Alves et al. (2025) reported that the ability of spirulina to restore tight junction integrity may be linked to its antioxidant properties. However, in the present study, although dietary spirulina enhanced the mRNA expression of SOD2 in LPS-challenged pigs, this effect did not translate into improvements in the mRNA levels of tight junction proteins. This finding, relative to other studies, may be attributed to species-specific differences in gut physiology and immune responses or to the severity and type of challenge model used.

While research on the beneficial effects of dietary spirulina supplementation in pigs is limited, a study by Choi et al. (2019) demonstrated that applying a spirulina extract to mouse microglial cells *in vitro* downregulated the mRNA expression of pro-inflammatory cytokines, including IL1β, TNFα, and IL6. In a similar study (Piovan et al., 2021), the application of spirulina extract at physiologically tolerable concentrations reduced the expression of key inflammatory mediators, including TNFα, IL1β, and inducible nitric oxide synthase, in LPS-stimulated brain cells, indicating the anti-inflammatory roles of spirulina.

In the current study, dietary spirulina supplementation did not reverse the LPS-induced reductions in villus height, perimeter, and area in the jejunum of pigs. This contrasts with previous studies that have reported beneficial effects of spirulina on intestinal morphology under both healthy and immune-challenged conditions. For instance, in broiler chickens (Attia et al., 2023), dietary spirulina alleviated heat stress-induced gut damage, an effect partly attributed to its phenolic compounds that promote the proliferation of beneficial bacteria. Similarly, it has been demonstrated that modified nanoparticles derived from *Spirulina maxima* increased villus height and goblet cell density in mice, highlighting its potential to improve intestinal structure (Chandrarathna et al., 2020). However, the inability of dietary spirulina supplementation to restore intestinal morphology may be attributed to its possible role in prioritizing the control of systemic inflammation and reduction of oxidative stress over the immediate structural repair of LPS-induced intestinal damage. Indeed, previous studies have shown that spirulina can inhibit NF-κB activation and reduce LPS-induced inflammatory cytokine production (Ku et al., 2013; Chei et al., 2020). However, further research is needed to clarify the relationship between the anti-inflammatory effects of spirulina and the onset of intestinal tissue repair following an LPS challenge.

In conclusion, this study demonstrates dietary spirulina supplementation enhances antioxidant activity and mitigates intestinal inflammation in pigs during an LPS challenge. By reducing oxidative stress and suppressing inflammatory cytokine production, spirulina helps maintain intestinal homeostasis under an immune stress condition. Additionally, irrespective of LPS exposure, dietary spirulina improved intestinal barrier function by enhancing tight junction integrity, which is crucial for preventing gut permeability and maintaining overall gut health. Furthermore, dietary spirulina supplementation contributed to improved nutrient utilization. These findings highlight the potential of dietary spirulina as a functional feed additive to support gut health, immunity, and nutrient digestibility in pigs. Lastly, future research should further investigate the mechanisms driving these benefits and evaluate the long-term effects of dietary spirulina supplementation on the intestinal health and growth performance of pigs.

## Supplementary Material

skaf338_Supplementary_Data

## Data Availability

The data that support the findings of this study are available from the corresponding author upon reasonable request.

## Abbreviations

ADFI: average daily feed intake
ADG: average daily gain
ARE: antioxidant response elements
ATTD: apparent total tract digestibility
BW: body weight
CAT: catalase
CLD1: claudin 1
CP: crude protein
CRP: c-reactive protein
DE: digestible energy
DM: dry matter
G:F: gain-to-feed ratio
GAPDH: glyceraldehyde-3-phosphate dehydrogenase
GE: gross energy
GPX1: glutathione peroxidase 1
IL10: interleukin 10
IL1β: interleukin 1 beta
IL6: interleukin 6
Keap1: kelch-like ECH-associated protein 1
LPS: lipopolysaccharide
N: nitrogen
NF-κB: nuclear factor kappa B
Nrf2: nuclear factor erythroid 2–related factor 2
OCLN: occludin
PBS: phosphate buffered saline
ROS: reactive oxygen species
SLC2A1: solute carrier family 2 member 1
SLC2A5: solute carrier family 2 member 5
SOD: superoxide dismutase
SOD2: superoxide dismutase type 2
SP: spirulina
TNFα: tumor necrosis factor alpha
VH:CD: villus height-to-crypt depth ratio
ZO1: zonula occludens-1
