# Supplementary material for: Assessment of dietary spirulina supplementation on growth performance, nutrient digestibility, and intestinal health in lipopolysaccharide-challenged weanling pigs
Source: J Anim Sci. 2025 Sep 27;103:skaf338. doi: 10.1093/jas/skaf338 (PMC12569516; doi:10.1093/jas/skaf338)
Supplement: skaf338_Supplementary_Data [file skaf338_supplementary_data.docx]

**Table S1.** Sequence of primers used for the real-time PCR analysis.

| **Target Gene** | **Primer Sequence (5’ to 3’)** | **Temperature (˚C)** | **Accession number** |
| --- | --- | --- | --- |
| IL1β | F: CCAAAGAGGGACATGGAGAA | 55.7 | NM_001302388.2 |
|  | R: GGGCTTTTGTTCTGCTTGAG |  |  |
| IL6 | F: TCTGGGTTCAATCAGGAGACCTGC | 55.9 | NM_214399.1 |
|  | R: TGCACGGCCTCGACATTTCCC |  |  |
| TNFα | F: CGTCGCCCACGTTGTAGCCAAT | 56.0 | NM_214022.1 |
|  | R: GCCCATCTGTCGGCACCACC |  |  |
| IL10 | F: CCTGGAAGACGTAATGCCGA | 62.2 | NM_214041.1 |
|  | R: CACGGCCTTGCTCTTGTTTT |  |  |
| SLC2A1 | F: GCCCCCGCTTCCTGCTCAT | 57.5 | X17058.1 |
|  | R: GCCAGGGCCCACTTCAAAG |  |  |
| SLC2A5 | F: GCATCTCTACCGCCTGTGTTGG | 52.9 | XM_021072101 |
|  | R: GCTTCTTGAATGTCCTCCTCCTCTG |  |  |
| ZO1 | F: AAGCCCTAAGTTCAATCACAATCT | 59.5 | XM_003353439.2 |
|  | R: ATCAAACTCAGGAGGCGGC |  |  |
| CLD1 | F: TTTCCTCAATACAGGAGGGAAGC | 64.0 | NM_001244539.1 |
|  | R: CCCTCTCCCCACATTCGAG |  |  |
| OCLN | F: CTACTCGTCCAACGGGAAAG | 59.8 | NM_001163647.2 |
|  | R: ACGCCTCCAAGTTACCACTG |  |  |
| GPX1 | F: TACAGCCGTCGCTTTCTGAC | 58.3 | NM_214201.1 |
|  | R: CACTCTAGGCACTGCTAGGC |  |  |
| SOD2 | F: TTGTAGGAGCGCCGAATACC | 58.9 | NM_214127.2 |
|  | R: ACCTGAACAAGCCGCATTCA |  |  |
| GAPDH | F: GTTTGTGATGGGCGTGAAC | 55.7 | NM_001206359.1 |
|  | R: ATGGACCTGGGTCATGAGT |  |  |

Abbreviations: F = Forward primer; R = Reverse primer; IL1 beta = Interleukin 1 beta; IL6 = Interleukin 6; TNFα = Tumor necrosis factor alpha; IL10 = Interleukin 10; SLC2A1 = Solute carrier family 2 member 1; SLC2A5 = Solute carrier family 2 member 5; ZO1= Zonula occludens-1, CLD1 = Claudin 1; OCLN = Occludin; GPX1 = Glutathione peroxidase 1; SOD2 = Superoxide dismutase type 2; GAPDH = Glyceraldehyde-3-phosphate dehydrogenase.
